# Biocompatible Nanobioglass Reinforced Poly(ε-Caprolactone) Composites Synthesized via In Situ Ring Opening Polymerization

**DOI:** 10.3390/polym10040381

**Published:** 2018-04-01

**Authors:** Zoi Terzopoulou, Diana Baciu, Eleni Gounari, Theodore Steriotis, Georgia Charalambopoulou, Dimitrios Bikiaris

**Affiliations:** 1 Laboratory of Polymers Chemistry and Technology, Department of Chemistry, Aristotle University of Thessaloniki, GR54124 Thessaloniki, Greece; terzoe@gmail.com; 2 National Center for Scientific Research “Demokritos”, Ag. Paraskevi Attikis, Athens GR15341, Greece; dianabaciuro@yahoo.com (D.B.); t.steriotis@inn.demokritos.gr (T.S.); gchar@ipta.demokritos.gr (G.C.); 3 Biohellenika Biotechnology Company, Leoforos Georgikis Scholis 65, GR57001 Thessaloniki, Greece; eleni-790@hotmail.com

**Keywords:** poly(ε-caprolactone), bioglass, nanocomposites, ring opening polymerization, biomineralization

## Abstract

Poly(ε-caprolactone) (PCL) is a bioresorbable synthetic polyester widely studied as a biomaterial for tissue engineering and controlled release applications, but its low bioactivity and weak mechanical performance limits its applications. In this work, nanosized bioglasses with two different compositions (SiO_2_–CaO and SiO_2_–CaO–P_2_O_5_) were synthesized with a hydrothermal method, and each one was used as filler in the preparation of PCL nanocomposites via the in situ ring opening polymerization of ε-caprolactone. The effect of the addition of 0.5, 1 and 2.5 wt % of the nanofillers on the molecular weight, structural, mechanical and thermal properties of the polymer nanocomposites, as well as on their enzymatic hydrolysis rate, bioactivity and biocompatibility was systematically investigated. All nanocomposites exhibited higher molecular weight values in comparison with neat PCL, and mechanical properties were enhanced for the 0.5 and 1 wt % filler content, which was attributed to extensive interactions between the filler and the matrix, proving the superiority of in situ polymerization over solution mixing and melt compounding. Both bioglasses accelerated the enzymatic degradation of PCL and induced bioactivity, since apatite was formed on the surface of the nanocomposites after soaking in simulated body fluid. Finally, all samples were biocompatible as Wharton jelly-derived mesenchymal stem cells (WJ-MSCs) attached and proliferated on their surfaces.

## 1. Introduction

PCL is an aliphatic, biodegradable polyester that can be synthesized via the ring opening polymerization (ROP) of ε-caprolactone (ε-CL). Its degradation rate can range from several months to several years and depends on various factors, like molecular weight, crystallinity, degradation media, etc. PCL is an FDA-approved polymer [1] for hard and soft tissue applications, biocompatible and miscible with several polymers, easily processed and moulded, properties that led to a wide range of applications, especially in tissue engineering, drug delivery, and food packaging [2,3]. However, when it comes to biomedical applications, PCL does not possess the essential hydrophilicity, mechanical properties, or a fast enough degradation rate [1]. Since a single component polymeric material cannot meet all the required specifications, research is focusing on multi-component systems, mainly with the incorporation of inorganic nanofillers into biodegradable polymeric matrices [4]. Nanofillers have the ability to enhance mechanical and crystalline properties of polymers, modulate the biodegradation rates and to mimic the composition of tissues [5]. Additionally, when nanofillers contain certain elements that are also constituents of the inorganic phase of active tissues such as the bone, the final nanocomposite biomaterials have the ability to induce bone regeneration [6,7].

Nanocomposites that consist of a biopolymer and bioceramics present favorable mechanical properties especially as scaffolds for bone regeneration and other biomedical applications [8,9]. Indeed PCL/bioactive glass composites and nanocomposites can be used as coatings for metallic or ceramic scaffolds for bone regeneration [10], as a solution against high corrosion rates or low bioactivity [9,11,12,13], as root canal filling materials [8], or as bone scaffolds [14,15,16,17,18]. 

The in situ polymerization method for the fabrication of nanocomposites has been proven a quite effective technique for the exploitation of the intriguing properties of various nanofillers and subsequent improvement of the mechanical, thermal, electrical, gas barrier properties, especially when compared to the two other dominant methods of solvent casting and melt compounding [19,20,21]. In situ polymerization allows the formation of covalent bonds between the nanofillers and the polymeric matrix, leading to a stronger interface and better dispersion that affect positively the final properties of the nanocomposites [22,23]. Composites and nanocomposites with bioactive glasses are predominately synthesized with solution mixing, melt blending or electrospinning [24]. Despite the large number of publications on bionanocomposites containing nanosized bioactive glasses, to our knowledge there is no study that deals with the synthesis of nanobioglass-containing nanocomposites with the in situ polymerization method. The presence of metal oxides is expected to affect the ROP mechanism possibly by either interacting with the catalyst used and acting as initiators, or by forming covalent bonds during the propagation of the macromolecular chains and acting as chain extenders or crosslinking agents.

In a previous study of our group, 2.5 wt % of a micron-sized bioactive glass was incorporated in a poly(butylene succinate) (PBSu) matrix using the in situ polymerization method, which resulted in a composite material with lower molecular weight and inferior mechanical properties compared to neat PBSu due to the large size of the filler, the addition of which though accelerated the biodegradation rate of the matrix and induced bioactivity. Evidence of interactions between the filler and the matrix were found in FTIR spectra [25]. 

The main aim of the present work was to improve the mechanical and biological properties of PCL, by combining it with two types of nanosized this time bioglasses, namely SiO_2_–CaO and SiO_2_–CaO–P_2_O_5_, which were synthesized with a hydrothermal method. The nanobioglasses were used as nanofillers in the in situ ROP of ε-caprolactone (ε-CL) in various concentrations (0.5, 1 and 2.5 wt %) in the presence of Tin(II) 2-ethylhexanoate (TEH) as catalyst. The obtained nanocomposites were studied in terms of their morphological, structural, crystalline, thermal and mechanical properties. In addition, the effect of both nanofillers on the enzymatic hydrolysis rate and the in vitro bioactivity of the final hybrids was also investigated, along with Wharton jelly-derived mesenchymal stem cell’s (WJ-MSCs) viability studies. 

## 2. Materials and Methods

### 2.1. Materials

ε-Caprolactone (CL) monomer (purity 99%), Tin(II) 2-ethylhexanoate (TEH) catalyst (analytical grade), poly(ethylene glycol) (PEG) (average *M*_n_ 10,000 g/mol), tetraethyl orthosilicate (TEOS) (reagent grade 98%), triethyl phosphate (TEP) (purum >99.8%), calcium nitrate tetrahydrate Ca(NO_3_)_2_·4H_2_O) (ACS reagent 99%), cetyltrimethylammonium bromide (CTAB) and ethanol were purchased from Sigma-Aldrich chemical company (Saint Louis, MO, USA). Sodium hydrate pellets were supplied from Mallinckrodt Company (Staines-upon-Thames, UK). All the other reagents used were of analytical grade and purchased by Sigma-Aldrich. 

### 2.2. Synthesis of Binary and Ternary Nanobioglasses

Ordered mesoporous bioactive glass nanospheres, based on both binary (SiO_2_–CaO) and ternary (SiO_2_–CaO–P_2_O_5_) systems, were prepared through a hydrothermal method based on a slighltly modified protocol to what was previously described [26,27], by using PEG and CTAB, as non-ionic co-surfactant and cationic surfactant, respectively. Briefly, for the preparation of the ternary SiO_2_–CaO–P_2_O_5_ nanobioglass with composition 76% SiO_2_, 14%CaO and 10% P_2_O_5_ in mol percent (hereafter denoted as tBG), proper amounts of PEG and sodium hydrate were dissolved in distilled H_2_O under vigorous stirring, followed by the addition of CTAB. After stirring for 1 h at room temperature, Ca (NO_3_)_2_·4H_2_O, TEOS and TEP were also added to the mixture. This was kept at room temperature under vigorous stirring for 24 h and then transferred into 120 mL Teflon-lined autoclaves. The autoclaves were sealed and heated at 80 °C for 48 h and then allowed to cool down to room temperature naturally. The products were collected by filtration and washed several times with distilled H_2_O and ethanol, and dried at 100 °C overnight. Finally, the white powder obtained was calcined in air at 600 °C for 5 h with a heating rate of 9 °C/min. The same protocol, with the necessary adaptations, was also followed to obtain the binary bioglass system SiO_2_–CaO with molar composition 76% SiO_2_ and 24% CaO (hereafter denoted as bBG), without using TEP. 

### 2.3. Synthesis of PCL Nanocomposites

For the synthesis of PCL, ε-CL was dried over CaH_2_ and purified by distillation under reduced pressure prior to use. The bulk polymerization of ε-CL was carried out in a 250 mL round-bottom flask equipped with a mechanical stirrer and a vacuum apparatus. The catalyst TEH was added as a solution in toluene at a final concentration of 1 × 10^−4^ mole per mole of monomer. The polymerization mixture was de-gassed and purged with dry argon three times. The ROP reaction was carried out for 3 h at 190 °C, followed by increasing the reaction temperature from 210 to 240 °C over a period of 90 min. Unreacted monomer was removed through distillation by applying high vacuum (≈5 Pa) slowly, to avoid excessive foaming, over a time period of 15 min. Polymerization was terminated by rapid cooling to room temperature.

The same procedure was also followed for the preparation of PCL/bBG and PCL/tBG nanocomposites by in situ polymerization. bBG or tBG nanoparticles were dried at 100 °C for 24 h under vacuum prior to polymerization, and were added in the ε-CL monomer along with the proper amount of TEH. The mixture was sonicated initially for 2 min using a tip sonication apparatus, and then in a sonic bath for 15 min. Polymerization was then carried out. Nanocomposites containing 0.5, 1 and 2.5 wt % bBG and tBG have been prepared.

The obtained hybrid materials were afterwards hot pressed using an Otto Weber, Type PW 30 hydraulic press (Paul-Otto Weber GmbH, Remshalden, Germany) connected with an Omron E5AX Temperature Controller (Omron, Kyoto, Japan), at a temperature of 75 ± 5 °C, in order to prepare a variety of films with different thickness, appropriate for each characterization technique used, as described in Section 2.4.

### 2.4. Physicochemical Characterization

TEM experiments were carried out on a JEOL 2011 TEM (Jeol Ltd., Akishima, Japan) with a LaB6 filament and an accelerating voltage of 200 kV. The specimens were prepared by evaporating drops of bBG and tBG in ethanol suspensions deposited after sonication onto a carbon-coated lacy film supported on a 3 mm diameter, 300 mesh copper grid.

Fourier transform infrared spectroscopy (FTIR) spectra of the nanofillers were obtained using a Thermo Scientific Nicolet (Thermo Fisher Scientific, Waltham, MA, USA) 6700 FTIR spectrometer equipped with an attenuated total Reflectance (ATR) accessory. FTIR spectra of the nanocomposite samples were obtained using a PerkinElmer (PerkinElmer Corporation, Waltham MA, USA) FTIR spectrometer, model Spectrum One. The materials were in the form of thin films with thickness of approximately 15 mm. The FTIR spectra of these films were obtained in absorbance mode and in the spectral region of 400–4000 cm^−1^ using a resolution of 4 cm^−1^ and 64 co-added scans.

The powder X-ray diffraction (PXRD) patterns of the nanofillers were recorded on a Rigaku R-AXIS IV Imaging Plate Detector (Rigaku, Tokyo, Japan) mounted on a Rigaku RU-H3R (Rigaku, Tokyo, Japan) Rotating Copper Anode X-ray Generator (λ = 0.154 nm). 

The pore properties of the nanobioglasses were determined by the nitrogen adsorption/desorption measurements at 77 K using a volumetric gas adsorption analyser (AUTOSORB-1-MP, Quantachrome Instruments, Boynton Beach, FL, USA). Prior to measurement, the samples were appropriately outgassed (at 250 °C for 12 h) under high vacuum (10^−6^ mbar), while ultra-pure N_2_ was used. The BET area values were calculated by the Brunauer–Emmett–Teller method, following the BET consistency criteria. Pore size distributions were deduced by fitting the adsorption isotherms on the basis of a non-local density functional theory kernel developed for N_2_ at 77 K on silica materials with cylindrical pores. 

Size exclusion chromatography (SEC) experiments were carried out at 30 °C using a Spectra System PL 1000 pump, a Shodex RI 101 refractive index detector, and a Spectra System UV-1000 detector. Three mixed-C (Polymer Laboratories, with pores for efficient separation of molecules varying from 2000 to 4 × 10^6^ g/mol) columns were used, thermostated in a Lab Alliance column oven at 30 °C. THF, distilled over CaH_2_ and sodium, was the carrier solvent at a flow rate of 1 mL/min. The samples were prepared by dissolution in THF and filtering with Whatman^®^ Puradisc 25 syringe filters supplied by Sigma-Aldrich (Saint Louis, MO, USA). 

The morphology of the prepared nanocomposites before and after enzymatic hydrolysis was examined using a JEOL JMS-840A scanning electron microscope (SEM) equipped with an energy-dispersive X-ray Oxford ISIS 300 microanalytical system (Oxford Instruments, Tubney WoodsAbingdon, Oxfordshire, UK). All samples were coated with carbon black to avoid charging under the electron beam. 

Measurements of tensile mechanical properties of the prepared nanocomposites were performed on an Instron 3344 dynamometer (Norwood, MA, USA) in accordance with ASTM D638, using a crosshead speed of 50 mm/min. Dumb-bell-shaped tensile test specimens (central portions 5 × 0.5 mm thick, 22 mm gauge length) were cut in a Wallace cutting press and conditioned at 25 °C and 55–60% relative humidity for 48 h. The values of Young’s modulus, yield stress, elongation at break and tensile strength at the break point were determined. For the notched Izod impact tests, a Tinius Olsen apparatus was used under ASTM D256. The specimens were prepared in a similar way and conditioned as described above. At least five specimens were tested for each sample in order to derive the average values. 

Differential scanning calorimetry (DSC) measurements were performed with a Pyris-6 instrument (Perkin Elmer, Waltham, MA, USA) calibrated with Indium and Zinc standards, under N_2_ flow. For each measurement, 5–10 mg of the sample was placed in a sealed aluminum pan and heated from ambient temperature to 100 °C with a heating rate of 10 °C/min, and subsequently cooled to 10 °C with a cooling rate of 5 °C/min. The crystallinity fraction X_c_ % was calculated by taking into account that the theoretical heat of fusion Δ*Η*_m_ for 100% crystalline PCL is 135 J/g [28].

Thermogravimetric analysis was carried out with a Setsys 16/18 TG-DTA (Setaram Instrumentation, Caluire, France). Samples were placed in alumina crucibles and heated from ambient temperature to 620 °C at 20 °C/min using a 50 mL/min flow of N_2_; an empty alumina crucible was used as reference.

The apparent contact angle of water was measured by a contact angle analyzer (Kruss EasyDrop Standard, Hamburg, Germany). Contact angle was measured by gently placing a water droplet (5 μL) on the surface of the nanocomposite films. The presented values were averaged over at least five points for each sample. 

For the enzymatic degradation testing, the samples in the form of films with 3 × 3 cm in size and approximately 2 mm thickness, were placed in petri dishes containing phosphate buffer solution (pH 7.2) with 1 mg/mL pseudomonas Cepacia lipase. The petri dishes were then incubated at 36.6 ± 1 °C in an oven for several days while the media were replaced every 3 days. After a specific period of incubation (every 3 days), the films were removed from the petri dishes, washed with distilled water, dried under vacuum and weighed until constant weight. The degree of enzymatic hydrolysis was estimated from the mass loss of the samples. The morphology of the films during hydrolysis was studied with SEM and crystallinity was calculated with DSC measurements, as described above. 

For the in vitro biomineralization experiments, the nanocomposites in the form of films were soaked in simulated body fluid (SBF) and incubated at 37 °C in closed falcon tubes for 14 days in order to assess their bioactivity. The ionic concentrations in the SBF solution were nearly equal to those in human body blood plasma, and the solution was buffered at pH 7.4 with trimethanol-aminomethane. After the immersion test, the films were removed and washed three times with deionized water to remove adsorbed minerals. The films were dried under vacuum and characterized by SEM using the conditions described previously.

### 2.5. Cell Cultures

#### 2.5.1. Isolation, Cultivation and Genetic Modification of Wharton Jelly-Derived Mesenchymal Stem Cells (WJ-MSCs)

35 cm of umbilical cord blood were collected after the parents endorsed consent in a sterilized box. Following a normal saline wash and a mild cut up with a lancet, an overnight lysis with 4 mg/mL collagenase and 2 mg/mL dispasecontained in PBS in a stirring incubator was performed. The next day, the mixture was filtered through a 70 μm Filter Unit and was subsequently centrifuged in 850 g for 10 min in room temperature. The pellet was resuspended in DMEM media supplemented with 10% FBS + 1% Penicillin/Streptomycin (DMEM full medium) and was then plated in culture flasks for 72 h until the full cell adherence in a 37 °C incubator with 5% CO_2_. Between the 4–5 passage a Pt2-Venus-neo mediated nucleofection was performed. Μore precisely, 4 × 10^5^ cells were mixed with 7.5 μg of plasmid DNA SB100X transposaseandpT2-Venus-neo transposon expression plasmids (1:10 ratio) and were put to electroporation according to the manufacturer’s instructions (Lonzabio). The cells were then plated in one well of a 6-well plate in the presence of DMEM full medium until reaching a 90% confluency, whereas 100 mg/mL G418 was added for the selection of the genetically modified WJ-MSCs.

#### 2.5.2. Sterilization of the Materials and WJ-SCs Plating

All PCL films, upon cutting in 9 × 9 mm dimension, were sterilized in gradually reduced ethanol concentrations (100%–70%–50%) and after being washed three times with distilled-deionized H_2_O were let to air dry for 4 h under sterile conditions. Fibrin glue was prepared after the blood sampling of a healthy volunteer donor. 15 μL of fibrin glue per film were placed in the bottom of a 24-well plate and the materials were seeded using a sterile pincher from above by applying minimal manual pressure and were let to air dry overnight under sterile conditions. 

WJ-MSCs were detached using Trypsin-EDTA 1× in PBS and were counted in a Neubauer cell counting chamber. 3 × 10^5^ cells were resuspended in DMEM full medium and were subsequently placed above the films of each condition. Upon air drying for 4 h in the incubator 1 mL DMEM full medium was added per well for the culture initiation.

#### 2.5.3. 3-[4,5-Dimethylthiazole-2-yl]-2,5-diphenyltetrazolium Bromide (MTT) Assay

In order to assess the cytotoxicity levels of films, the MTT assay was performed (Sigma-Aldrich) 24 h after the initial cell plating. Briefly, after the medium removal from the wells, MTT reactant was introduced in a ratio of 1:10 in DMEM culture medium and was followed by a 4 h incubation in 37 °C with 5% CΟ_2_. Upon the removal of the MTT, 1 mL/well of DMSO was introduced for one additional hour of incubation in the same conditions. The reduction of MTT was counted at wavelengths 570 and 630 nm (Perkin Elmer).

#### 2.5.4. Observation in a Fluorescence Microscope

The observation of the cells above the materials was performed under a fluorescence HBO 50 mercury lamp as well as reflectors with fluorescence filter (excitation 488 nm, emission 509 nm), while the program for downloading and editing the photos was the Fluorescence Lite software module of AxioVision LE (Carl Zeiss, Oberkochen, Germany).

## 3. Results and Discussion

### 3.1. Characterization of the Nanofillers

**Figure 1:**
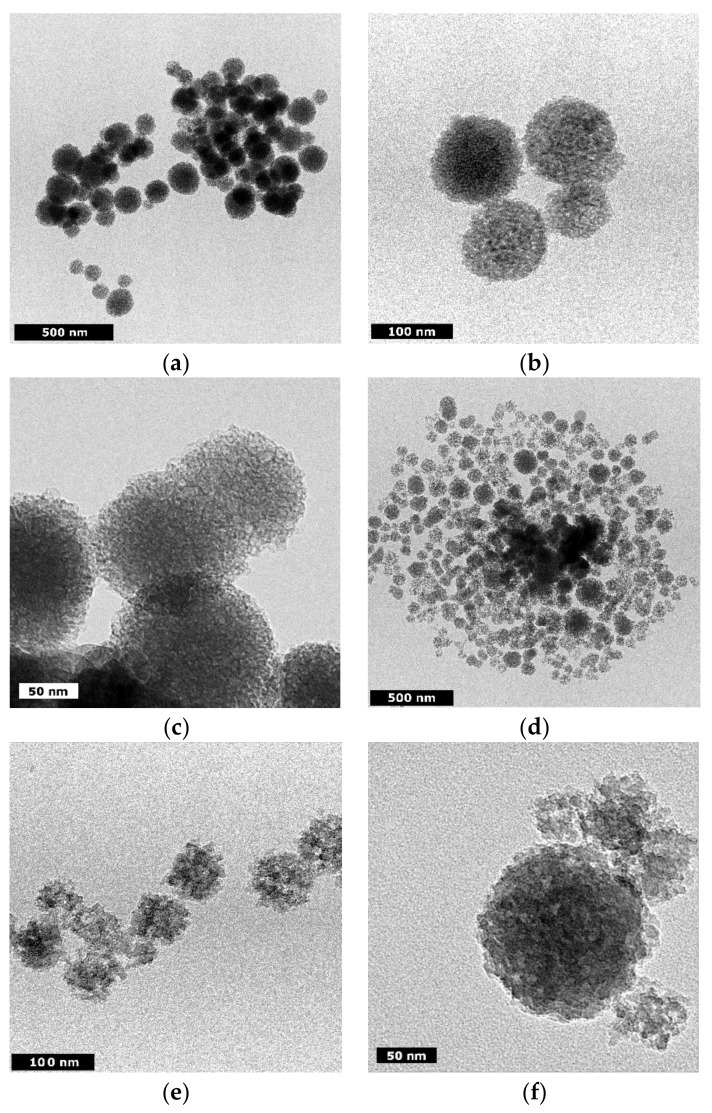
TEM micrographs of (**a**–**c**) bBG and (**d**–**f**) tBG.

TEM micrographs of bBG and tBG nanofillers (Figure 1) show that the hydrothermal method employed and the calcination at 600 °C leads in both cases to the formation of porous spherical nanoparticles with size ranging at 100–150 nm for bBG and 80–130 nm for tBG. According to literature [29], the use of PEG, comprising a relatively new synthetic approach, may improve the dispersion of the nanoparticles, maintain their stability and/or control their shape. Furthermore, PEG may act as a pore-forming agent due to its thermal decomposition [30].

**Figure 2:**
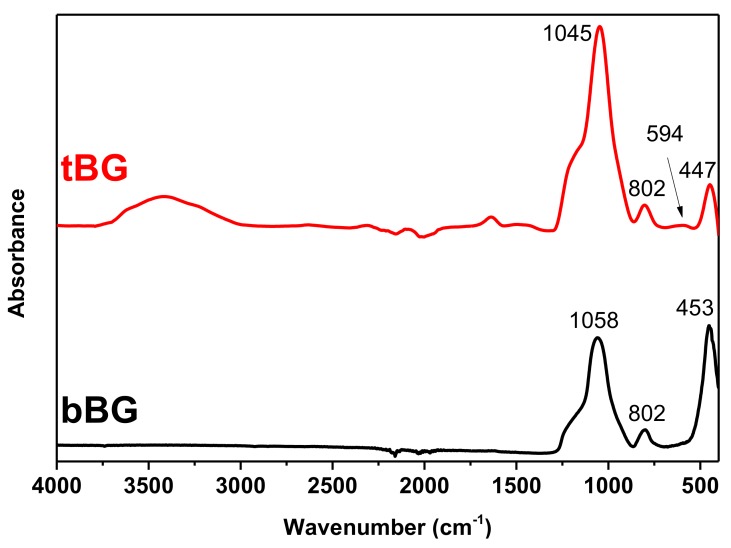
FTIR spectra of bBG and tBG.

The FTIR spectra of both nanobioglasses are presented in Figure 2. bBG exhibited three peaks at 453, 802 and 1058 cm^−1^, respectively. The peak at 453 cm^−1^ is assigned to the bending vibrations of the Si–O–Si and O–Si–O bonds [31,32]. The peak at 802 cm^−1^ corresponds to the stretching vibrations of the O–Si–Ο bonds [31,32,33] and the peak at 1058 cm^−1^ is attributed to the symmetric stretching vibration of the Si–Ο–Si bonds [32,33]. No peaks that can be assigned to organic matter have been observed, confirming the purity of the materials. Similar peaks were also observed for the tBG sample. In addition, the FTIR pattern of the tBG sample depicts a weak band at 594 cm^−1^ attributed to the asymmetric vibration of PO_4_^3−^ [34]. The peak at 3400 cm^−1^ is attributed to –OH groups [35].

**Figure 3:**
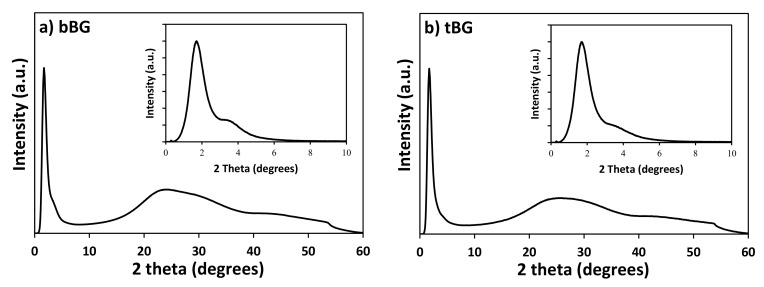
Wide-angle and low-angle XRD patterns of (**a**) bBG and (**b**) tBG.

The wide-angle PXRD patterns of the calcined bBG and tBG materials depicted in Figure 3 confirm the amorphous nature of these materials, in agreement with previous studies [36]. In the low angle region (Figure 3, insets) the PXRD patterns of both bBG and tBG show a strong peak at 2θ = 1.7° and an additional broad shoulder centered at about 2θ = 3.4° (which is weaker though for tBG), indicating that the pores exhibit to a certain degree long range ordering [37,38] that partially deteriorates for the ternary system. 

**Figure 4:**
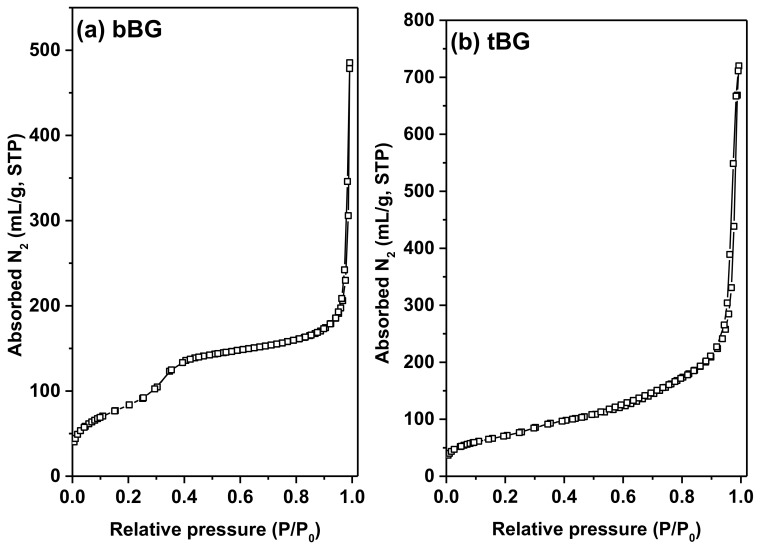
Adsorption-desorption isotherms of (**a**) bBG and (**b**) tBG nanobioglasses.

**Figure 5:**
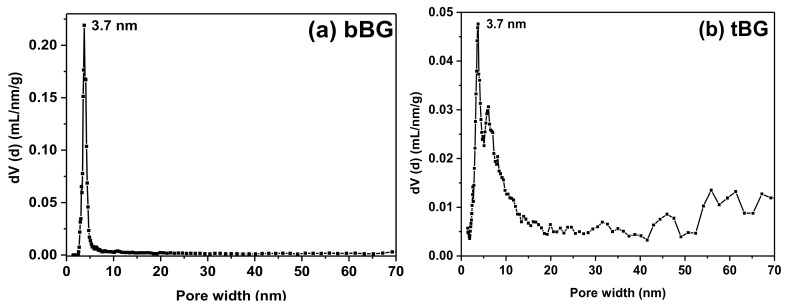
Pore size distribution curves of (**a**) bBG and (**b**) tBG.

The pore properties of the calcined bBG and tBG samples were determined by the analysis of N_2_ adsorption/desorption isotherms at 77 K (Figure 4), which are of type IV (according to IUPAC classification), characteristic of mesoporous materials. In both cases significant meso- to macro-porosity is also evident at high relative pressures where a significant increase of the amount adsorbed is recorded; this volume is attributed to the pore space created by the packing of bioglass nanospheres. Overall bBG was found to have a BET area of 305 m^2^/g and a total pore volume of 0.37 cm^3^/g (the latter calculated at *P*/*P*_0_ = 0.98); the respective values for tBG were 256 m^2^/g and 0.67 cm^3^/g (at *P*/*P*_0_ = 0.98). In addition, both materials showed a mean pore size of ca. 3.7 nm according to the deduced pore size distributions shown in Figure 5.

**Figure 6:**
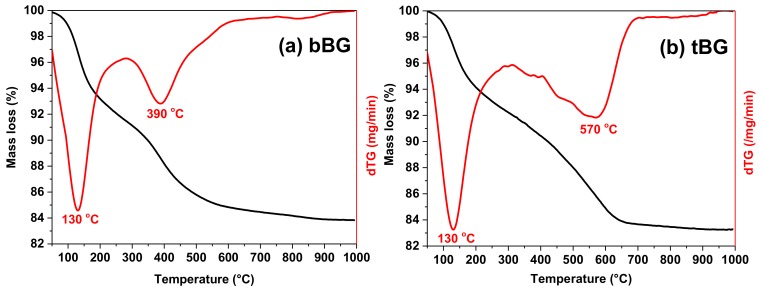
TGA and DTG thermograms of (**a**) bBG and (**b**) tBG.

The thermal stability of bBG and tBG after calcination was confirmed with TGA and the results are presented in Figure 6. Both nanobioglasses exhibit two main weight loss steps. The first one with a *T*_max_ at 130 °C corresponds to loss of about 4% physically absorbed water. The second step of mass loss of bBG occurs in the temperature range 250–500 °C with *T*_max_ at 390 °C, while tBG loses about 10% of its mass in the range 250–600 °C with a main *T*_max_ at 570 °C. This may be caused from the release of water formed from the condensation of Si–OH and P–OH groups of the nanobioglasses [39]. In general, both glasses are very thermally stable as expected, and lose 16% of their initial mass when heated to 1000 °C.

### 3.2. Synthesis and Characterization of PCL Nanocomposites

#### 3.2.1. Synthesis of PCL Nanocomposites via In Situ ROP

PCL is mainly synthesized by the ROP of the cyclic lactone, ε-CL, with a wide range of initiators/catalysts. TEH is one of the most commonly used catalysts since it is highly effective and approved by FDA for use in food packaging due to its low toxicity [40,41,42]. TEH catalyzes the ROP of CL in the presence of a nucleophilic compound, usually an alcohol that allows the formation of the active species which are tin alkoxides [43,44], however small amounts of polar impurities or traces of moisture in the polymerization mixture are enough to initiate the polymerization. No alcohol was used in the present case, in order to study the effect of the nanofillers on the polymerization reaction. The active species initiate the polymerization of ε-CL via a coordination-insertion mechanism that enables the production of stereoregular polymers while controlling the molecular weight and the molecular weight distribution [45]. Metal alkoxides can trigger side reactions like inter- and intramolecular transterifications that lead to an increase of the polydispersity index (PDI), however, the effectiveness of TEH, as well as its commercial availability, easy handling, and good solubility render it an attractive catalyst in ROP procedures [2,37].

Molecular weight values and PDI as measured by GPC are presented in Table 1, where $\bar{M}n$ is the number average molecular weight, $\bar{M}w$ the weight average molecular weight and $\bar{M}\nu$ the viscosity average molecular weight. The presence of the fillers affected the reaction, resulting in variations of the final molecular weight values. Neat PCL has a $\bar{M}$**w** of 71,912 g/mol, which is considered a satisfactory value. After the incorporation of the nanofillers, the nanocomposite polyesters exhibited higher $\bar{M}$**w** values that increase when the filler content is increased, up to 96,177 g/mol, suggesting that both nanobioglasses might act as chain extenders. 

To our knowledge, nanosized bioactive glass/polymer composites have not been previously synthesized with the in situ polymerization method. In a previous work of our group, a poly(butylene succinate) (PBSu) nanocomposite with 2.5 wt % microsized bioglass was synthesized with the in situ esterification/polycondensation method, and its molecular weight was found to be significantly decreased in comparison with neat PBSu [25]. This reduction was attributed to the hindering of water or monomer evaporation during the melt polycondensation stage, caused by the filler. In this work, nanoscale bioactive glasses were used instead, and the opposite effect on the $\bar{M}$**w** was noticed, indicating that particle size plays an important role in the properties of in situ synthesized composites. Hydroxyapatite (HA) fillers have been found to act as weak initiators during the ROP of ε-CL in the presence of TEH catalyst, resulting in polymers with lower molecular weights since the hydroxyl groups of HA lead to a higher concentration of chain-ends [46]. On the contrary, the presence of nanosized SiO_2_ nanoparticles has been found to increase the molecular weights of polyesters, especially in low concentrations, and this was attributed to the ability of silanol groups to react with alcohols and act as chain extenders [47,48,49]. MgO nanocrystals were also found able to increase the molecular weight of PLA at 0.01 wt % nanofiller content since according to the authors, the end-capping of polymeric chains by MgO was counterbalanced by the total number of polymeric chains on each nanocrystal resulting in a larger hydrodynamic volume [50]. 

#### 3.2.2. Morphological Characterization

**Figure 7:**
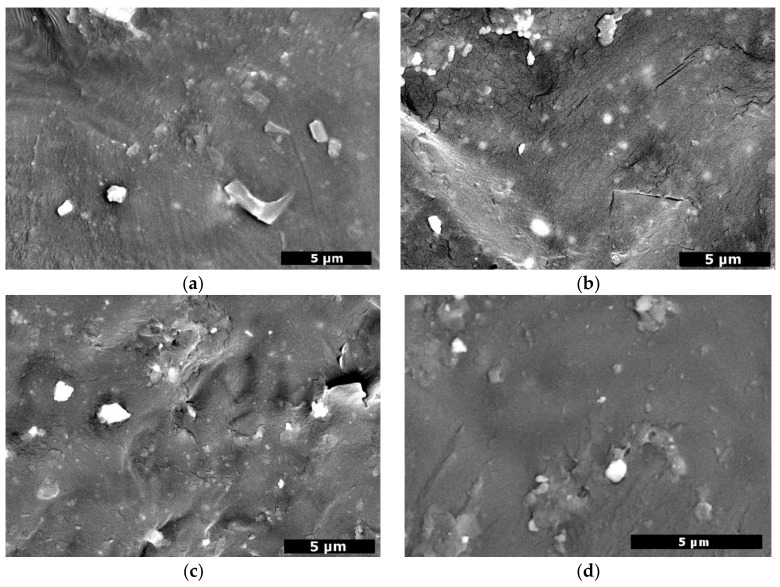

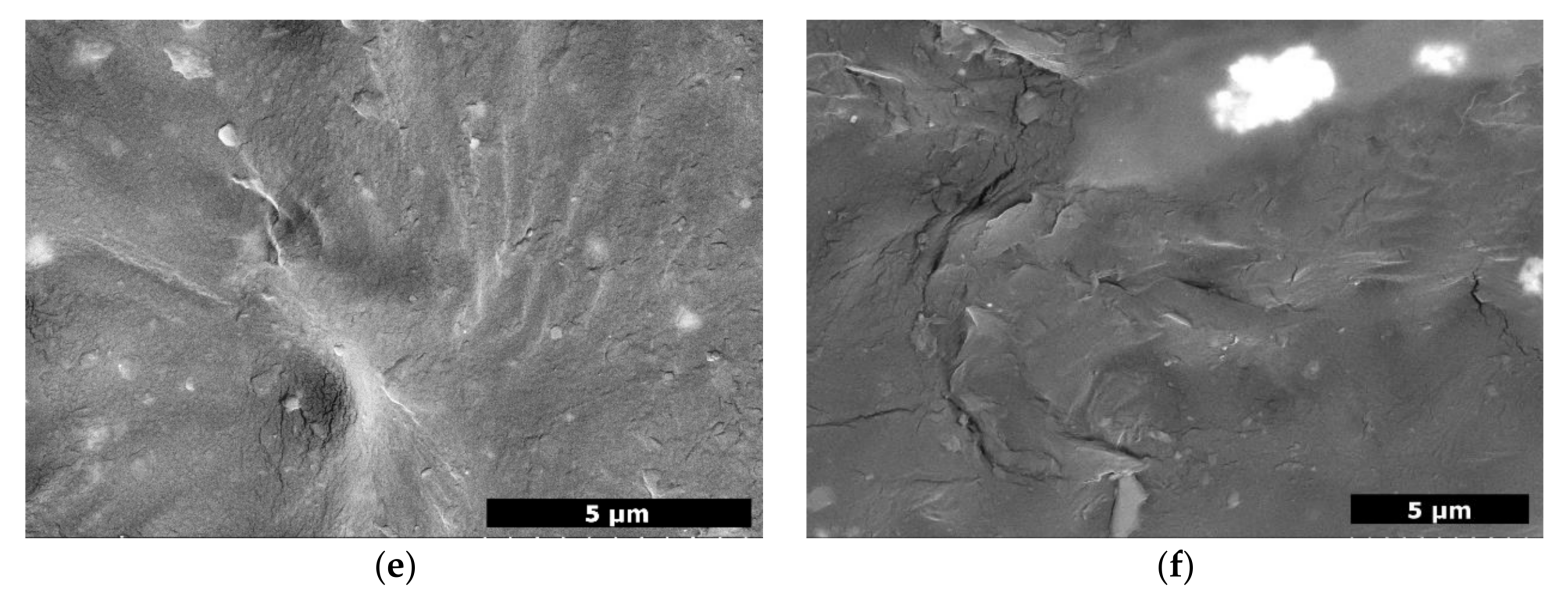
Backscattered SEM micrographs of (**a**) PCL/bBG 0.5%, (**b**) PCL/bBG 1.0%, (**c**) PCL/bBG 2.5%, (**d**) PCL/tBG 0.5%, (**e**) PCL/tBG 1.0%, (**f**) PCL/tBG 2.5%.

The surface of the prepared nanocomposite films was examined by SEM (Figure 7). Neat PCL was smooth without any particular texture, while all nanocomposites had irregular surfaces and the nanofillers imparted them with significant roughness, in agreement with other studies [51]. This is attributed to the spherical ‘peaks’ the fillers form, creating micro and nanotopography. Concerning the dispersion, the nanobioglasses under the surface detected by backscattering seem well dispersed, while the ones on top of the films form aggregates that are greater in number and bigger in size as concentration increases.

#### 3.2.3. Structural Characterization

**Figure 8:**
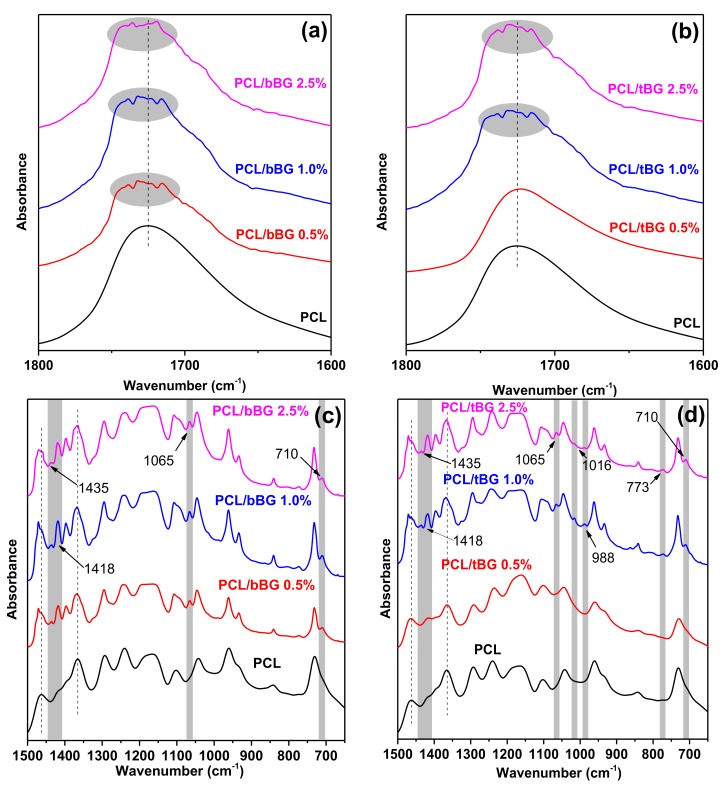
FTIR spectra of (**a**) PCL/bBG nanocomposites, (**b**) PCL/tBG nanocomposites and (**c**,**d**) zoomed in the region 1500–650 cm^−1^.

**Figure A1:**
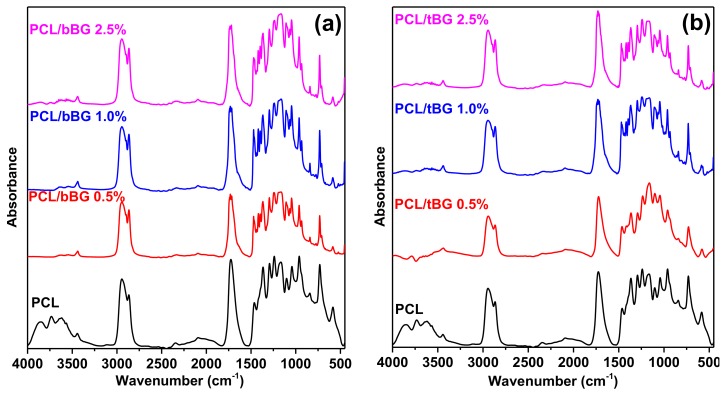
FTIR spectra of (**a**) PCL/bBG and (**b**) PCL/tBG nanocomposites.

FTIR spectroscopy was used to evaluate the chemical structure of the synthesized polyesters. The recorded spectra are presented in Figure A1. Neat PCL shows characteristic peaks at 1725 cm^−1^ due to the stretching vibration of the >C=O bond, at 2921 cm^−1^ and 2864 cm^−1^ due to the symmetric and asymmetric stretching vibrations of the >CH_2_ groups, at 1108 cm^−1^ and 1238 cm^−1^ due to the symmetric and asymmetric stretching vibrations of the C–O–C bridge of the ester group, at 1463 cm^−1^ due to the bending vibrations of the O–H of the end carboxyl groups and at 1365 cm^−1^ due to the O–H of the hydroxyl end group bending vibration [15,52]. Additionally, a broad peak that consists of 4 peaks of medium intensity appears in the region 4000–3200 cm^−1^. These peaks that appear at 3850, 3732, 3620, and 3440 cm^−1^ are generally derived from hydroxyl group related vibrations, and are attributed to stretching of the hydroxyl end groups, stretching of intermolecularly hydrogen bonded hydroxyls, stretching of the carboxylic end group hydroxyls, and stretching of intramolecularly hydrogen bonded hydroxyls. All nanocomposites exhibit the basic peaks of the PCL structure; however, some differences can be noticed. The first major difference that can be spotted in the spectra of the nanocomposites in Figure A1 is the significant reduction of the intensity of the peaks in the region 4000–3200 cm^−1^, which is in accordance with the molecular weight values reported above, since the higher the molecular weight, the smaller the amount of hydroxyl and carboxyl end groups in the macromolecules [53]. Besides molecular weight increase, carboxyl end groups could have formed bonds with the –OH groups of the bioglasses, created by absorbed moisture, resulting in diminishing of the corresponding peaks [54,55]. Regarding the peaks of the carbonyl group, they split into multiple peaks in the spectra of the nanocomposites as seen in Figure 8a,b. The peak at 1725 cm^−1^ corresponds to the free C=O groups of PCL, while the emergence of the new peaks in the carbonyl region is evidence of hydrogen bonding or the presence of carbonyls with different secondary groups off the carbonyl carbon [25,56]. After zooming in the region 1500–650 cm^−1^ (Figure 8c,d), it can be noticed that the main peak of bBG and tBG powders observed at 1045 and 1058 cm^−1^ respectively are shifted to 1065 cm^−1^ in the nanocomposites, which is another indication of interactions of the Si–O–Si moiety of the fillers with the polymer. Additionally, new peaks emerge for both bBG and tBG nanocomposites at 710 cm^−1^ because of Si–O–Si vibrations, at 1418 cm^−1^ possibly due to carbonated calcium ions that are not incorporated in the glass network [57], and at 1435 cm^−1^ which arises from absorbed moisture. PCL/tBG nanocomposites have more FTIR peaks than PCL/bBG at 773, 988, and 1016 cm^−1^ because of the additional phosphate group of tBG’s composition that can be attributed to the stretching vibration of HPO_4_^2−^ [58], the symmetric stretching of PO_4_^3−^, and the antisymmetric stretching of PO_4_^3−^, respectively [58]. 

Shifting of FTIR peaks suggests the presence of interactions between nanofillers and polymeric matrices. Such alterations in FTIR spectra have been observed in PET/SiO_2_ nanocomposites and were attributed to the action of the nanofillers as chain extenders. When added in high concentration (5 wt %), SiO_2_ nanoparticles resulted in a decrease of the molecular weight due to extensive crosslinking reactions, but in lower concentrations a substantial increase was observed [59]. A similar phenomenon was also observed for PBSu/SiO_2_ nanocomposites, where filler contents up to 2.5 wt % increased the molecular weight of the matrix due to covalent bonding that was confirmed through FTIR and ^13^C NMR spectroscopy [47], as well as for poly(ethylene succinate)/SiO_2_ nanocomposites where higher molecular weight values were attributed to covalent crosslinking and branching [48]. Therefore, the observed alterations of the FTIR spectra of the PCL nanocomposites with nanobioglasses suggest the presence of extensive interactions between the polymer and the fillers that can be responsible for the increased molecular weights presented above. 

**Figure 9:**
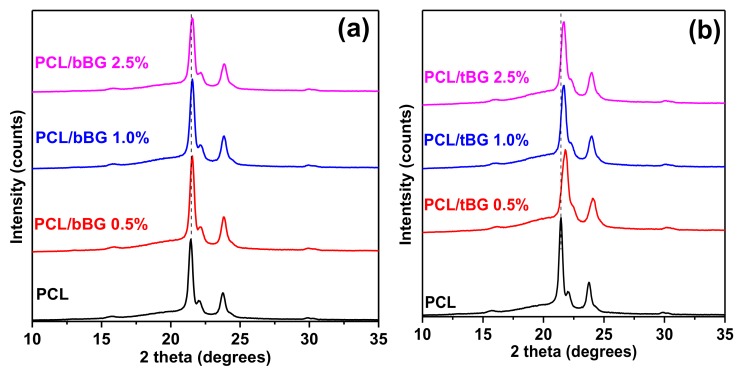
X-ray diffraction patterns of (**a**) PCL/bBG nanocomposites and (**b**) PCL/tBG nanocomposites.

The recorded WAXD patterns of PCL and its nanocomposites are presented in Figure 9. PCL shows 3 crystalline peaks at 2θ = 21.45°, 22° and 23.8° that correspond to the (110), (111) and (200) planes of the orthorhombic unit cell [50,52]. These peaks were still present after the incorporation of both nanofillers, therefore the crystal structure of PCL remained unaffected*.* However, slight shifts were observed especially in the case of tBG, which may indicate alterations in the crystallite sizes of the respective nanocomposites. 

#### 3.2.4. Mechanical Properties

**Figure 10:**
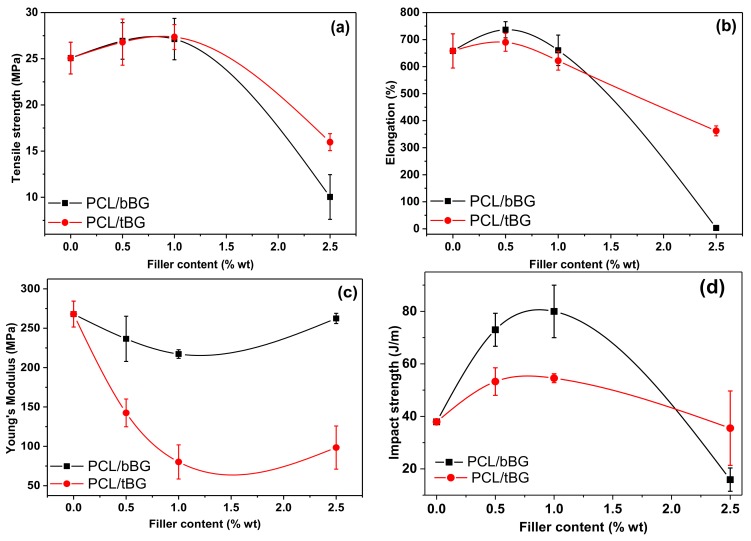
Effect of nanofiller type and content on (**a**) tensile stress at break, (**b**) elongation at break, (**c**) Young’s Modulus and (**d**) impact strength of PCL nanocomposites. The lines are just a guide to the eye.

The effect of the two nanobioglasses on the mechanical properties of PCL was evaluated by measuring the tensile properties as well as the impact strength of the nanocomposites. The resulting values are presented in Figure 10. Neat PCL has a tensile stress at break value of 25.07 ± 1.72 MPa which is slightly increased in the presence of 0.5 and 1 wt % fillers, up to 27.13 ± 2.24 MPa for 1 wt % bBG and 27.35 ± 1.35 MPa for 1 wt % tBG, respectively. However, increasing the filler loading to 2.5 wt % leads to a gradual decrease of tensile strength, since the fillers formed aggregates that act as premature mechanical failure points [15,60,61,62]. A similar trend is observed for elongation, which was measured 658.09 ± 63.45% for PCL and increased to 736.43 ± 29.91% and 690.11 ± 33.20% after the incorporation of 0.5 wt % bBG and 0.5 wt % tBG respectively, and decreased at higher concentrations. This enhancement could be attributed to the higher molecular weight of the nanocomposites, the satisfactory dispersion of the nanofillers in PCL and the formation of strong interactions between the two of them. In another study, Bioglass^®^ particles were found able to reinforce the mechanical properties of poly(glycerol sebacate) networks through the formation of calcium ion bridges with the free carboxylic acid groups of the polymer [60]. Young’s modulus values were found decrease for all nanocomposites, meaning they were less stiff and more susceptible to elastic deformation, except for the nanocomposite PCL/bBG 2.5 wt % that had almost the same modulus value with neat PCL. Young’s modulus usually increases in the presence of stiff glass fillers in composite and nanocomposite materials prepared by melt blending or solution mixing [15,17,61]. In our case, the stiffness decreased but it is not considered an important disadvantage since both tensile stress at break and elongation values increased for the two lowest filler contents. 

Impact strength of PCL was also altered by the two fillers, which increased with both bBG and tBG 0.5 and 1 wt %, from 38 J/m up to 80 J/m for the sample PCL/bBG 1 wt % (Figure 10d). The increase is larger for bBG particles [51]. Bioactive glass particles of diameter 40 nm reduced tensile and impact strength of PLLA nanocomposites, in contents 2.5 to 20 wt %, so it can be concluded that enhancement of mechanical properties by these fillers can be achieved in very low concentrations, even lower than 2.5 wt % [62].

#### 3.2.5. Thermal Characterization

Table 2 shows the *T*_m_, *T*_c_, and X_c_ values of PCL and its nanocomposites. PCL has a *T*_m_ = 65.4 °C, which is slightly increased for the nanocomposites containing 0.5 and 1 wt % nanobioglasses, and reduced in the presence of 2.5 wt % nanofillers. *T*_c_ follows the same trend. X_c_ increased for all nanocomposites, in proportion with filler content, which could also have contributed to the enhancement of the mechanical properties. The higher melting point values are related to the higher molecular weights of the nanocomposites. Nanofillers can act as heterogeneous nucleation sites, therefore increasing *T*_c_ during cooling as well as overall crystallinity. The increase in crystallinity could also have an impact on the improved mechanical properties measured (Section 3.2.4). 

It has been reported that bioglass particles of several sizes reduced the *T*_m_ and X_c_ of PCL in concentration 21% vol and was attributed to the formation of more defective crystals and hindering of chain mobility [63]. *T*_m_ reduction was found independent from micro-sized the composition of the glasses [64]. In contrast with larger bioactive glass particles, nanosized bBG and tBG had the opposite effect on thermal properties due to their small size that allows them to impart an increase in the molecular weight values and enhance crystallinity. The synthesis method also seems to have played a role in the enhancement of crystallinity and *T*_m_. In our previous study, it was found that the stronger the interactions in PCL nanocomposites synthesized with the ROP of ε-CL, the more enhanced its crystallization properties [52]. Liu et al. noticed that when incorporating bioactive glass nanoparticles in poly(*L*-lactide) (PLLA) with the solution mixing method, crystallization rates were retarded, but after grafting the nanoparticles with PLLA prior to nanocomposite preparation, nucleation and crystallization rates were improved [62]. Thus, the strong interactions created during the in situ ROP synthesis of nanocomposites positively affect the crystallization properties of PCL, proving it a more effective method for the improvement of a polymer’s properties with the incorporation of nanofillers. 

Crystallinity is one of the parameters that affects cellular responses, among chemical structure, topography, hydrophilicity and morphology. Increased crystallinity values in PCL/PGA copolymers were found to enhance fibroblast adhesion and proliferation, because it can cause topographical changes and induce rigidity, but the opposite effect was observed for osteoblasts [15,65,66]. 

**Figure A2:**
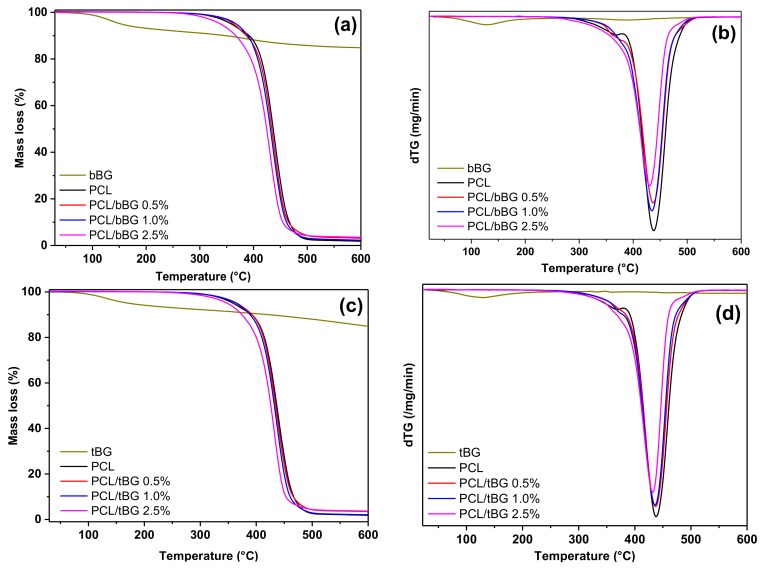
(**a**,**c**) Mass loss curves and (**b**,**d**) DTG curves of PCL/bBG and PCL/tBG nanocomposites.

TGA and DTG thermograms of PCL and its nanocomposites are presented in in Figure A2, and the characteristic thermal degradation temperatures *T*_d,2%wt_ at which the sample loses 2% of its weight and *T*_max_ where degradation occurs with the highest rate are presented in Table 3. The mass loss curves reveal that degradation occurs in one step that starts at about 350 °C. The *T*_max_ of the degradation of PCL is at 438.2 °C and it slightly decreases for all the nanocomposites. The higher the filler content, the less thermally stable the nanocomposite. While both nanofillers make PCL less thermally stable, tBG seems to affect it more. 

Micro-sized bioglass particles have been reported to dramatically decrease the thermal stability of biodegradable polymers, because of their Si–O–Si bonds that are terminated with hydroxyl groups on their surface. These groups, in the presence of moisture, can be dissociated with Si–O^−^ and associated with Ca^2+^ counterions that could catalyze chain scission [67]. In fact, the reduction of *T*_max_ by the presence of 2 vol % and 15 vol % bioglass in PLLA was about 100 °C, rendering the filler’s modification prior to incorporation necessary [67,68]. Blaker et al., based on FTIR spectra observations, suggested that the reaction that occurs between bioactive glasses and polyesters at elevated temperatures and catalyzes the degradation is: RSiO^−^ + R’COOR’’ → RSiOR’’ + R’CO^2−^ [69]. The deterioration of thermal stability in this work, is insignificant compared to what has been reported in other publications. This can be attributed to (a) the preparation method of the nanocomposites, which is not solution casting but in situ polymerization and (b) the smaller size of the fillers. These two factors may have given rise to interactions between the surface silanol groups of the nanofillers and the end-groups of the polymer chains, reducing their total amount of available free Si–OH groups that can catalyze the chain scission of PCL during thermal degradation. According to this hypothesis, tBG accelerates the degradation of PCL more than bBG due to its more hydrophilic character (see Section 3.2.6) and consequently the presence of more silanol groups on its surface.

#### 3.2.6 Wettability and Enzymatic Hydrolysis

Hydrophilicity is an important parameter for biomaterials because it can affect their degradation rate, cell attachment and proliferation. Contact angle values are presented in Table 4. All nanocomposites were slightly more hydrophilic than neat PCL, which had a contact angle value of 81.6**°** ± 0.68, that decreases while increasing filler content. This can be attributed to the inherent hydrophilicity of the filler particles [51,61,70]. Also, it is observed that tBG increased hydrophilicity more compared to bBG, likely because it contains the additional hydrophilic P_2_O_5_ groups rather than only SiO_2_ and CaO like its binary counterpart. This hypothesis can be supported by the FTIR spectra of Figure 2 where in the case of tBG a peak that corresponds to water related vibrations is present. This peak is absent in the spectrum of bBG, therefore it can be related to enhanced hydrophilicity, since both nanobioglasses were pretreated in the same manner before obtaining the spectra (drying under vacuum and storing in desiccator). 

**Figure 11:**
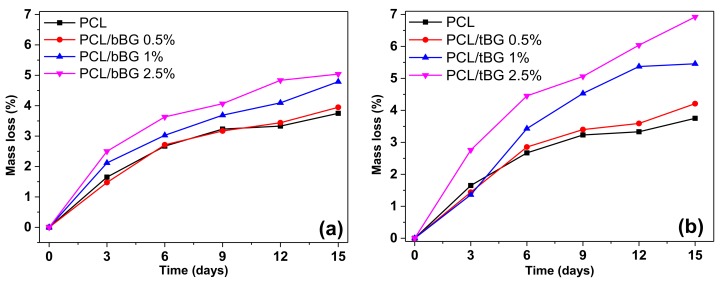
Plots of mass loss vs time of enzymatic degradation of (**a**) PCL/bBG and (**b**) PCL/tBG.

The effect of the nanobioglasses on the degradation rate of PCL was studied with measuring its weight loss after soaking in a solution of the lipase *P. cepacia* in phosphate buffer, as shown in Figure 11. Neat PCL is a hydrophobic polyester that degrades slowly, however like all degradable polyesters, its biodegradation rate can be controlled by the incorporation of nanofillers [5,71]. Indeed, all nanocomposites show an increased rate of mass loss that depends on the concentration of the two nanobioglasses. The higher the content of the hydrophilic fillers, the greater the mass loss of the films. As concluded by the contact angle measurements, all nanocomposites are more hydrophilic than neat PCL, making the diffusion of water along with the enzyme in the bulk material easier and finally turning PCL nanocomposites more susceptible to enzymatic degradation. It is also obvious that the PCL/tBG nanocomposites lose a bigger percentage of their weight compared to the PCL/bBG nanocomposites, which is in agreement with the contact angle values that suggest that tBG is more hydrophilic than bBG because of the presence of the phosphate groups in its structure. 

It has been reported in the literature that nanosized bioactive glass particles containing SiO_2_, CaΟ and P_2_O_5_ accelerated the degradation of PCL because of their hydrophilicity that accelerates the diffusion of the degradation media into the bulk material, and their own degradation along with the polymeric matrix [17,61]. 

**Figure 12:**
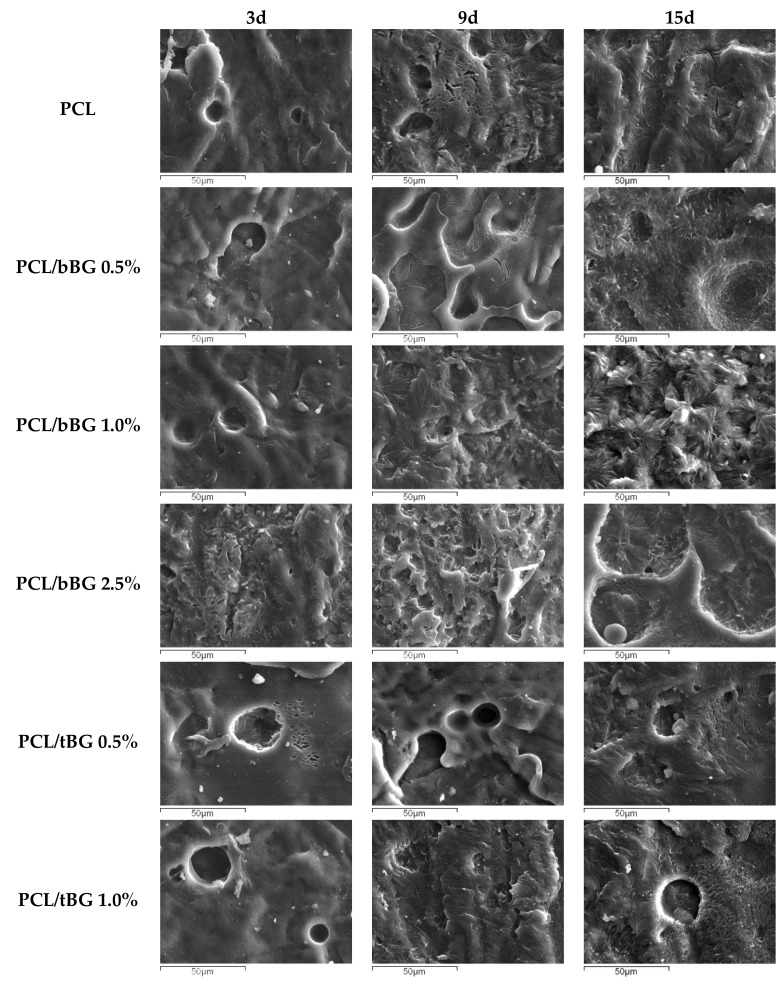

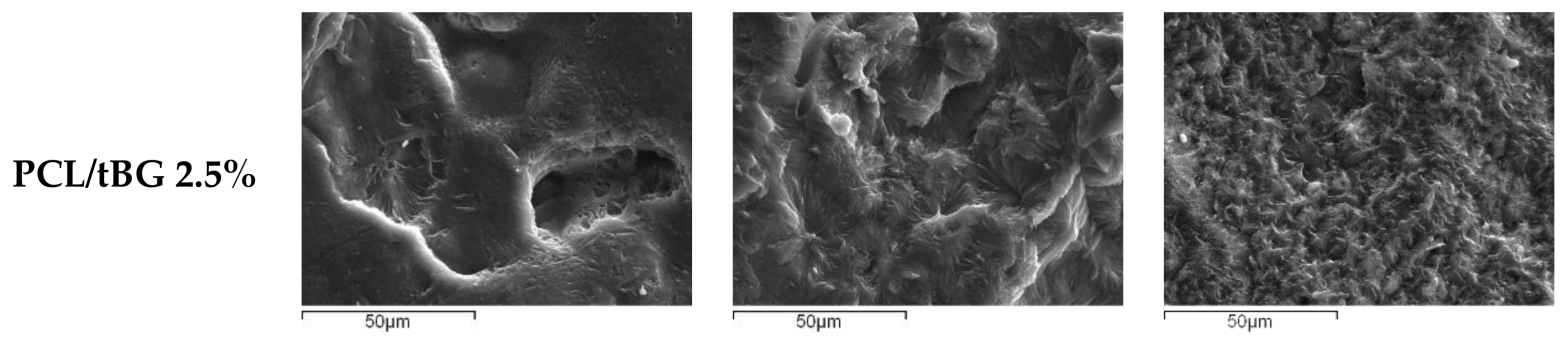
SEM micrographs of PCL and its nanocomposites after 3, 6 and 9 days of enzymatic degradation.

**Figure A3:**
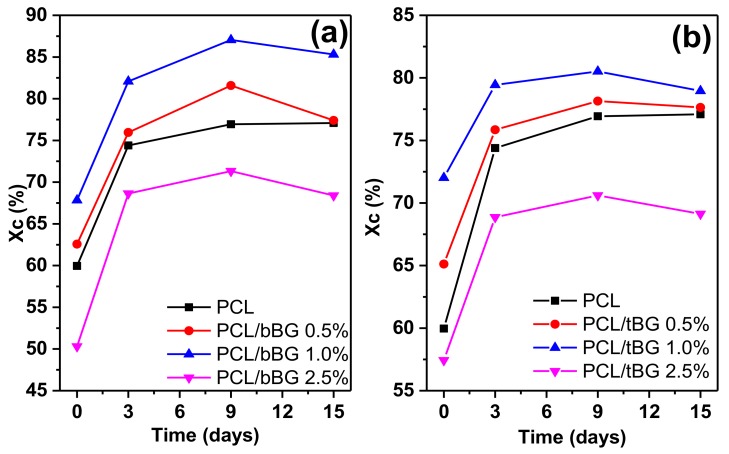
Changes in the crystallinity of (**a**) PCL/bBG and (**b**) PCL/tBG nanocomposites during enzymatic degradation (calculated by DSC, heating with rate 10 °C).

The surface of the films after enzymatic degradation was studied with SEM and the resulting micrographs are presented in Figure 12. After 3 days of soaking, a few holes appear on the surface of neat PCL, and as the degradation proceeds in 9 days, it becomes rougher. At 15 days morphologies that witness the presence of crystals appear, suggesting that hydrolysis occurs first in the amorphous parts and after that crystalline regions are exposed on the surface [5,72,73]. A similar trend is observed for all nanocomposites as well. Also, as nanofiller content increases, the irregularities of the films are more intense, which is in agreement with the weight loss measurements. The hypothesis that amorphous regions degrade first was further studied with the calculation of X_c_ of the films after enzymatic hydrolysis and the results are presented in Figure A3. Indeed, X_c_ values increase during the first 9 days in agreement with the observations from the SEM micrographs and decrease slightly after 15 days of soaking. Similar conclusions have been reported in the literature, where the crystallinity of PCL and its composites with bioglasses was found increased after 45 weeks of incubation in ultrapure water [15], and after incubation for 14 days in SBF [64]. 

#### 3.2.7 In Vitro Bioactivity

**Figure 13:**
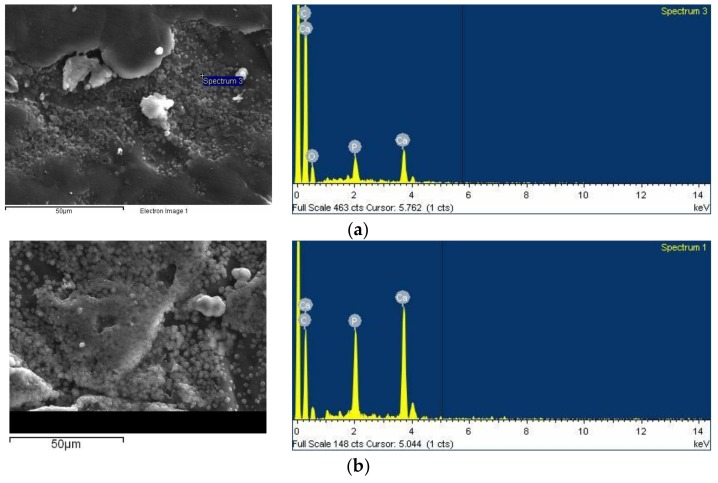
SEM micrographs and EDX spectra of (**a**) PCL/bBG 0.5% and (**b**) PCL/tBG 0.5% after 14 days of soaking in SBF.

Apatite mineralization is essential in the formation of bioactive interfaces between biomaterials and tissues, since it can promote osteoblast proliferation and differentiation [74]. Bioactivity is one of the most attractive features of bioglasses, since their incorporation in bioinert polymeric materials can make them bioactive [17,64]. The in vitro bioactivity of the nanocomposites was tested by incubation in SBF for 14 days and consequent examination of the surface morphology with SEM micrographs and EDX spectra. Figure 13 shows the results for the samples PCL/bBG 0.5% and PCL/tBG 0.5%. Both surfaces were found to be covered with spherical precipitates, especially on the imperfections of the films, unlike pure PCL which was found to be bioinert. The nanocomposites containing 1% and 2.5% bBG and tBG had fewer, smaller particles on their surface and are not presented for brevity. These formations were identified as apatite through the EDX spectra that showed peaks corresponding to Ca and P. The Ca/P ratio for PCL/bBG 0.5% surface was calculated 1.58 ± 0.7 and for PCL/tBG 0.5% 1.61 ± 0.4, which are close to the composition of stoichiometric apatite. The absence of large apatite crystals from the nanocomposites with 1 and 2.5 wt % filler could originate from the presence of aggregates rather than finely dispersed nanoparticles. Small bioglass particles have been found to cause faster formation of apatite compared with micron-sized particles in several studies [51,63,70,75,76]. 

#### 3.2.8 Adhesion and Proliferation of WJ-SCs

**Figure 14:**
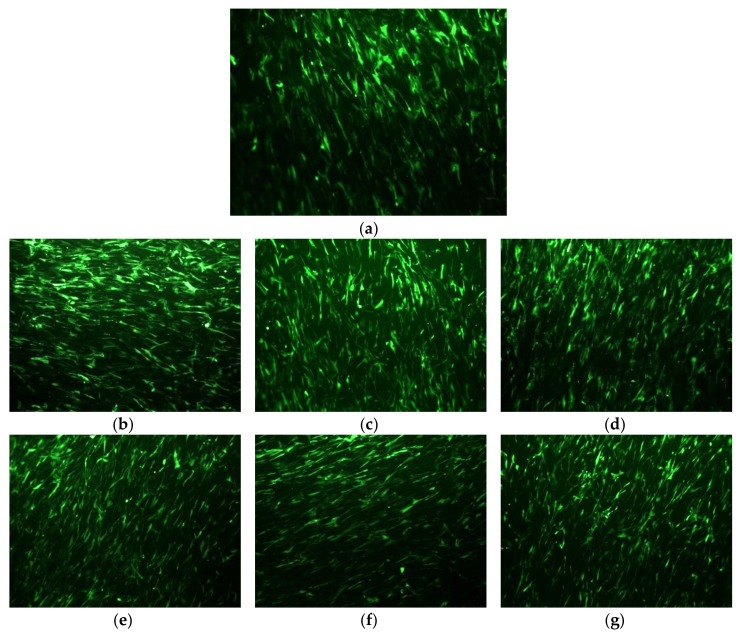
Genetically modified WJ-SCs on (**a**) PCL, (**b**) PCL/bBG 0.5%, (**c**) PCL/bBG 1%, (**d**) PCL/bBG 2.5%, (**e**) PCL/tBG 0.5%, (**f**) PCL/tBG 1.0% and (**g**) PCL/tBG 2.5%.

The biocompatibility of PCL and its nanocomposites with bBG and tBG was evaluated by seeding WJ-SCs on the surface of the materials. After 24 h, the viability of the cells was evaluated with fluorescence microscopy images, as presented in Figure 14. 

**Figure 15:**
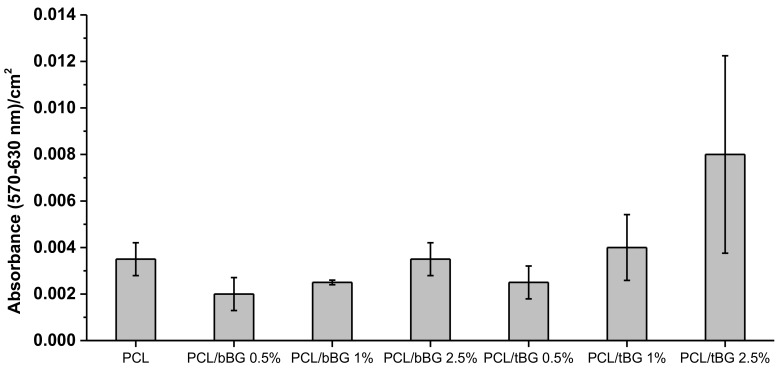
MTT assay results of WJ-SCs cells after seeding for 24 h on PCL and its nanocomposites.

PCL and all its nanocomposites displayed no significant toxicity after 24 h of the cultivation of WJ-SCs on their surfaces (Figure 15). The cell viability was confirmed by the morphological observation of WJ-SCs on the films with fluorescence microscopy, as presented in Figure 14. The viable cells appear as bright green spots and it is clear that they adhered and grew on all samples. MTT assay results of Figure 15 confirm the fluorescence microscopy findings. The performed t-test proved that the metabolic activity of all the nanocomposites was higher than the 50% of the metabolic activity of neat PCL, indicating their biocompatibility [25]. Additionally, metabolic activity increases while increasing each nanofiller’s content, suggesting their positive effect on cellular response, because of their high hydrophilicity, surface roughness and release of ions from their structure that affects gene expression and various cellular processes [54,61]. 

## 4. Conclusions

Nanocomposites of PCL with binary and ternary nanosized bioglasses were successfully prepared with the in situ ROP of ε-CL. The resulting nanocomposites had improved molecular weight values, thermal properties and mechanical properties in low concentrations. The improvement was attributed to the formation of extensive interactions between the fillers and the matrix during ROP. The nanocomposites were more hydrophilic than neat PCL, more susceptible to enzymatic hydrolysis, bioactive and biocompatible. In conclusion, both nanofillers were able to positively affect the properties of PCL, in very small filler contents, that requires necessary improvements when considered as a polymer for biomedical applications. However, ternary bioglass (SiO_2_–CaO–P_2_O_5_) due to the existence of P_2_O_5_ can give better in vitro bioactivity and metabolic activity on Wharton jelly-derived mesenchymal stem cells.

## Figures and Tables

**Table 1:** Molecular weight and PDI values measured by GPC.

Sample	$\bar{M}$n	$\bar{M}$w	$\bar{M}$ν	PDI
PCL	48,400	71,900	68,300	1.49
PCL/bBG 0.5%	53,900	83,900	78,700	1.55
PCL/bBG 1.0%	59,200	90,300	85,300	1.53
PCL/bBG 2.5%	61,900	88,900	84,600	1.44
PCL/tBG 0.5%	49,900	83,300	77,900	1.67
PCL/tBG 1.0%	51,400	84,500	78,800	1.64
PCL/tBG 2.5%	61,200	96,200	90,300	1.57

**Table 2:** Thermal characteristics of PCL and its nanocomposites as measured by DSC.

Sample	*T*_m_ (°C)	*T*_c_ (°C)	X_c_ (%)
PCL	65.4	31.9	59.96
PCL/bBG 0.5%	66.4	32.1	62.57
PCL/bBG 1.0%	67.1	32.4	67.83
PCL/bBG 2.5%	64.8	31.4	50.31
PCL/tBG 0.5%	66.4	32.3	65.12
PCL/tBG 1.0%	66.8	32.3	72.02
PCL/tBG 2.5%	65.1	30.7	57.43

**Table 3:** Characteristic temperatures of thermal degradation of PCL and its nanocomposites.

Sample	*T*_d,2%wt_ (°C)	*T*_max_ (°C)
PCL	329.9	438.2
PCL/bBG 0.5%	333.6	436.9
PCL/bBG 1.0%	335.8	434.8
PCL/bBG 2.5%	304.9	430.0
PCL/tBG 0.5%	330.1	436.9
PCL/tBG 1.0%	330.0	436.0
PCL/tBG 2.5%	311.8	431.9

**Table 4:** Water contact angle values of PCL and its nanocomposites.

Sample	Contact angle (°)
PCL	85.3 ± 0.68
PCL/bBG 0.5%	81.7 ± 0.3
PCL/bBG 1.0%	75.7 ± 0.41
PCL/bBG 2.5%	73.2 ± 0.3
PCL/tBG 0.5%	80 ± 2.87
PCL/tBG 1.0%	74.1 ± 0.48
PCL/tBG 2.5%	71.8 ± 0.21
